# Comparing Information Metrics for a Coupled Ornstein–Uhlenbeck Process

**DOI:** 10.3390/e21080775

**Published:** 2019-08-08

**Authors:** James Heseltine, Eun-jin Kim

**Affiliations:** School of Mathematics and Statistics, University of Sheffield, Sheffield S3 7RH, UK

**Keywords:** stochastic processes, Langevin equation, Fokker–Planck equation, information length, Fisher information, metrics, O-U process, probability density function

## Abstract

It is often the case when studying complex dynamical systems that a statistical formulation can provide the greatest insight into the underlying dynamics. When discussing the behavior of such a system which is evolving in time, it is useful to have the notion of a metric between two given states. A popular measure of information change in a system under perturbation has been the relative entropy of the states, as this notion allows us to quantify the difference between states of a system at different times. In this paper, we investigate the relaxation problem given by a single and coupled Ornstein–Uhlenbeck (O-U) process and compare the information length with entropy-based metrics (relative entropy, Jensen divergence) as well as others. By measuring the total information length in the long time limit, we show that it is only the information length that preserves the linear geometry of the O-U process. In the coupled O-U process, the information length is shown to be capable of detecting changes in both components of the system even when other metrics would detect almost nothing in one of the components. We show in detail that the information length is sensitive to the evolution of subsystems.

## 1. Introduction

Describing many natural systems statistically can give great insight into the system’s dynamics, when uncertainty or degrees of freedom are too high to do otherwise. Measures of information change can be particularly useful in understanding the evolution of a system under perturbation, or comparing data (e.g., see [1]). Here, by information, we specifically refer to a measurable, statistical difference between the states of a system, defined by probability density functions (PDFs), and avoid any of the more diaphanous definitions of the term. The statistical difference can be quantified by assigning a metric to probability, which then endows a stochastic system with a geometric structure. Previously, different metrics (e.g., Refs. [2,3,4,5,6,7,8,9,10] have been considered depending on the question of interest.

A popular measure of the information change in a system would be entropy, which measures the uncertainty or ‘disorder’ of the system. More specifically, it is a measure of the number of states that are accessible from the current state. Comparing entropy at different times gives a measure of the difference in information for the system, called the relative entropy. We can use this relative entropy as a metric. Another example is the Wasserstein metric, which was used to optimize transport cost in the optimal transport problem [4,6]; for Gaussian PDFs, the Wasserstein metric is defined in the product space consisting of Euclidean and positive symmetric matrices for the mean and variance, respectively (e.g., see [2]). The link between the Fisher information [8] and the Wasserstein distance was made in [6] where the integral of the Fisher information [8] along the Ornstein–Uhlenbeck semigroup was shown to be the same as the Wasserstein distance. Furthermore [1] stated that relative entropy was the integral of Fisher information along the same path.

However, the way in which the relative entropy has mostly been used in the past lacks a sense of locality to a metric of the system as it focuses on quantifying the difference between two given PDFs, for instance, PDFs at time $t_{1}$ and $t_{2}$. As a result, they are independent of the intermediate PDFs between time $t_{1}$ and $t_{2}$ (the history/path of a system), and thus can only inform us about changes which affect the overall structure of the system. The work of [11] was, in part, a search for a disequilibrium component for a statistical complexity measure (SCM). In short, an SCM is a measure of both the ‘order’ and ‘disorder’ of a system, which can help to reveal hidden structures of a disordered system. They proposed several metrics ‘disorder’ or disequilibrium component of the SCM.

In this paper we compare several of the proposed metrics of [11] with the information length L [12,13,14,15,16,17,18,19,20]. The information length, proportional to the time integral of the square root of the infinitesimal relative entropy, depends on the intermediate states between $t=t_{1}$ and $t=t_{2}$ and is thus a Lagrangian measure. Also, the formulation of the information length allows us to measure local change for the system in time. $L_{\infty }$, the total information length over the entire evolution $t=t_{1}=0$ and $t=t_{2}\to \infty$, was shown to be useful to quantify the proximity of any initial PDF to a final attractor of a dynamical system. For instance, for the Ornstein–Uhlenbeck process (O-U) [16,18], $L_{\infty }$ was shown to take its minimum value at the stable equilibrium point and increase linearly with the mean position of an initial PDF from the stable equilibrium point. This linear dependence manifests that the information length preserves the linear geometry of the underlying Gaussian process. In this paper, we will show that this linear relation is lost for other metrics (e.g., relative entropy, Jensen divergence). Note that for a chaotic attractor, $L_{\infty }$ varies sensitively with the mean position of a narrow initial PDF, taking its minimum value at the most unstable point [21]. This sensitive dependence of $L_{\infty }$ on the initial PDF is similar to a Lyapunov exponent.

We note that the O-U is a prototypical relaxation problem and can be particularly useful to study, as its attractor provides a natural equilibrium state. It can model many stochastic systems which relax to a stable equilibrium. The solution to this process is Gaussian, and so has ‘nice’ properties of analytical tractability, permitting us to perform detailed investigation under the change of parameters. We first compare different metrics for a single O-U process then move to a coupled O-U process. The O-U process is a well studied model, though less so for the coupled system. Our focus is to compare different metrics and to see if the information length may be more revealing the behavior of the components of the coupled system, as well as the overall system. The remainder of this paper is organized as follows. Section 2 provides the definition of different metrics. Section 3 is devoted to the discussion of a single O-U process. Section 4 provides analytical solutions to the coupled O-U process and Section 5 compares different metrics for the coupled O-U process. Conclusions are found in Section 6. In Appendix A, we present how to solve the Fokker–Planck equation(s) numerically by using a second-order accuracy method in time and compare analytical results with numerical results. Appendix B comments on the Langevin equation for our coupled O-U process.

## 2. Information Length and Other Metrics

We consider a PDF P(x,t) for a stochastic variable *x* in the following.

### 2.1. Information Length

The information length L(t) between time 0 and *t* is given by

(1) $$L\left(t\right)=\int_{0}^{t}\frac{dt^{\prime }}{\tau (t^{\prime })}=\int_{0}^{t}dt^{\prime }\sqrt{\int_{-\infty }^{\infty }dx{\left(\frac{\partial P(x,t^{\prime })}{\partial t^{\prime }}\right)}^{2}\frac{1}{P(x,t^{\prime })}},$$

where $\frac{1}{{\left|\tau \left(t\right)\right|}^{2}}$ is the second moment given by,

(2) $$\frac{1}{{\left|\tau \left(t\right)\right|}^{2}}=\int_{-\infty }^{\infty }dx{\left(\frac{\partial P(x,t)}{\partial t}\right)}^{2}\frac{1}{P(x,t)}.$$

Here, τ(t) has the unit of time while L has no dimension. The parameter τ(t) is the characteristic timescale of the system, and quantifies the correlation time for the system [15]. Hence, $\frac{1}{\tau (t)}$ is the rate of change of the information in time. Integrating $\frac{1}{\tau (t)}$ over [0,t] gives the total number of statistically different states that a system passes through in time. We note that L quantifies the information change in time through the root-mean-squared fluctuating energy, using the second moment of the partial derivative with respect to time. When the parameters governing a PDF are known, the information length L(t) can be written in terms of the Fisher information metric [8,12,15].

As noted in Section 1, L(t) is a Lagrangian quantity and has the property of being a local measure, being sensitive to how P(x,t) evolves at different *x* in time. In comparison with entropy which is independent of the spatial (*x*) gradient of a PDF, it is this property that may elevate L(t) above entropy in revealing micro-scale interactions within a system.

The discrete version of Equations (1) and (2) are as follows:

(3) $$\begin{array}{ccc} L_{n} & = & h\sum_{i=0}^{n}\frac{1}{\tau_{i}}, \end{array}$$

(4) $$\begin{array}{ccc} \frac{1}{\tau_{i}^{2}} & = & \frac{s}{h^{2}}\sum_{j}P_{i}^{j}{\left[\ln\left(P_{i+1}^{j}\right)-\ln\left(P_{i}^{j}\right)\right]}^{2}. \end{array}$$

Here, *i* and *j* represent the discrete time and spatial point, respectively; $P_{i}^{j}$ is the discrete version of P(x,t), h=t/n is the time step while *s* is the spatial step.

### 2.2. Other Metrics

Here, we list the metrics taken from [11]. We will calculate each metric relative to the initial state PDF P(x,0) at t=0 in order to compare their time-evolution with that of the information length. That is, the metrics below are based on comparing two PDFs P(x,0) and P(x,t). For convenience, the metrics are given both for the continuous process and discrete approximation (that is used for numerical calculation) by using i,j as an index representing time and space, respectively and *s* as the spatial step, as above. The reference probability P(x,0) [$P_{0}$] will be the initial PDF while P(x,t) [$P_{i}$] is the PDF at time *t* [*i*].

#### 2.2.1. Euclidean norm

(5) $$\begin{array}{ccc} ||P\left(x,t\right)-{P\left(x,0\right)||}^{2} & = & \int dx\;{\left[P(x,0)-P(x,t)\right]}^{2},\\ \left|\right|P_{0}-P_{i}{\left|\right|}^{2} & = & s\sum_{j}{\left[P_{0}^{j}-P_{i}^{j}\right]}^{2}. \end{array}$$

Applying our standard notion of distance in Euclidean space seems like a natural extension. However, it quickly becomes apparent that the statistical space of stochastic systems is rarely well described by Euclidean metrics. Mostly included here as a base case, whilst this formulation seems appealing, it does not yield illuminating details about the disequilibrium of the system. We will use it as an example of a poor measure of information change.

#### 2.2.2. Wootters’ Distance

(6) $$\begin{array}{ccc} W[(P(x,0),(P(x,t)] & = & \cos^{-1}\left[\int dx\;{\left[P\left(x,0\right)\right]}^{\frac{1}{2}}{\left[P\left(x,t\right)\right]}^{\frac{1}{2}}\right],\\ W[P_{0},P_{i}] & = & \cos^{-1}\left[s\sum_{j}{\left[P_{0}^{j}\right]}^{\frac{1}{2}}{\left[P_{i}^{j}\right]}^{\frac{1}{2}}\right]. \end{array}$$

This metric, as the notion of statistical distance itself, originates in quantum information theory [9]. However, as quantum information theory is purely statistical in formulation, it can be applied to any systems defined by a PDF. Fundamentally, this metric is based on the principle that any finite number of measurements on a stochastic system will yield results that may not be exactly the same as underlying probability distributions. It would be impossible to distinguish 2 states whose real underlying probabilities are different less than a typical fluctuation of the error of measurement. This intrinsically defines a resolution for the system. The Wootters’ distance was shown to be a monotone transformation of the Hellinger distance [22].

#### 2.2.3. Kullback-Leibler Relative Entropy

(7) $$\begin{array}{ccc} K(P(x,0)|P(x,t)) & = & \int dx\;P\left(x,0\right)\ln\left(\frac{P(x,0)}{P(x,t)}\right),\\ K[P_{0}|P_{i}] & = & s\sum_{j}P_{0}^{j}\log\left(\frac{P_{0}^{j}}{P_{i}^{j}}\right). \end{array}$$

Kullback–Leibler relative entropy was first introduced by Solomon Kullback and Richard Leibler [10], and sometimes is referred to as the Kullback–Leibler divergence. It represents a measure of the difference between a probability distribution and some other reference probability distribution. Whilst a useful tool, it is not strictly a metric as it does not satisfy the triangle inequality. It is however used in the definition of some other quantities, such as the mutual information of two co-varying random variables, and the Jensen divergence.

#### 2.2.4. Jensen Divergence

(8) $$\begin{array}{ccc} J(P(x,0)|P(x,t)) & = & \frac{1}{2}\left[K(P(x,0)|P(x,t))+K(P(x,t)|P(x,0))\right],\\ J[P_{0}|P_{i}] & = & \frac{1}{2}\left[K\left[P_{0}|P_{i}\right]+K\left[P_{i}|P_{0}\right]\right]. \end{array}$$

The Jensen divergence is simply the symmetric version of the Kullback–Leibler divergence. Often it is referred to as the Jensen distance, and the square root of this quantity can be shown to be a metric [11], which can allow us to examine the statistical geometry of the system. The Jensen divergence is the mutual information of a random variable *x*, with a mixture distribution from P(x,0) and P(x,t), and a binary indicator variable used to build the distribution. In other words it is a measure of the mutual dependence of *x* on the way you construct the mixture, and thus quantifies the amount of information difference between the two distributions.

## 3. The O-U Process

The one-dimensional O-U process is based on the Langevin equation

(9) $$\dot{x}=-\gamma \left(x-\mu \right)+\xi ,$$

where *x* is the stochastic variable (e.g., position, velocity, etc.), γ is the damping constant, μ is the position of the attractor for the system, and ξ is a δ-correlated, Gaussian-distributed stochastic forcing, i.e.,

(10) $$\langle \xi \left(t_{1}\right)\xi \left(t_{2}\right)\rangle =2D\delta \left(t_{1}-t_{2}\right),$$

where *D* is the strength of the stochastic forcing. The corresponding Fokker–Planck equation [23,24] is given by

(11) $$\frac{\partial P}{\partial t}=\frac{\partial }{\partial x}\left[\gamma \left(x-\mu \right)P\right]+D\frac{\partial^{2}P}{\partial x^{2}},$$

where the solution P=P(x,t) is the time-dependant PDF which describes the evolution of the system. It can be shown that the solution to Equation (11) is given by [13]

(12) $$P\left(x,t\right)=\sqrt{\frac{\beta }{\pi }}e^{-\beta {\left(x-\langle x\rangle \right)}^{2}},$$

where $\frac{1}{2\beta }=\frac{e^{-2\gamma t}}{2\beta_{0}}+\frac{D}{\gamma }\left(1-e^{-2\gamma t}\right)$ and $\langle x\rangle =x_{0}e^{-\gamma t}$, given the initial condition

(13) $$P\left(x,0\right)=\sqrt{\frac{\beta_{0}}{\pi }}e^{-\beta_{0}{\left(x-x_{0}\right)}^{2}},$$

where $\beta =1/2\langle {\left(x-\langle x\rangle \right)}^{2}$ and 〈x〉 in Equation (12) represent the inverse temperature and the mean value of *x*, respectively, and $\beta_{0}$ and $x_{0}$ in Equation (13) are the values of β and 〈x〉 at t=0, respectively.

### 3.1. Information Length

In [13], we showed that the information length L(t) for the O-U process is given by

(14) $$L=\frac{1}{\sqrt{2}}{\left[\ln\left(\frac{y-r}{y+r}\right)\right]}_{y_{i}}^{y_{f}}+\frac{\sqrt{2}}{r}\int_{y_{i}}^{y_{f}}\frac{qr-r^{2}}{y^{2}+qr-r^{2}}dy,$$

where $y=\sqrt{r^{2}+qT}$, $r=2\beta_{0}D-\gamma$, $q=\beta_{0}\gamma x_{0}^{2}$, and $T=\beta_{0}D\left(e^{2\gamma t}-1\right)+\gamma$. $y_{i}$ is *y* evaluated at the initial time (t=0 in our case) and $y_{f}$ is *y* evaluated at final time. $x_{0}$ is the initial mean position.

Let the integral in the last term of Equation (14) be *H*, and let r≠q≠0. Then Equation (14) can be written as

(15) $$L=\frac{1}{\sqrt{2}}{\left[\ln\left(\frac{y-r}{y+r}\right)\right]}_{y_{i}}^{y_{f}}+\frac{\sqrt{2}}{r}H,$$

where

(16) $$H=\left\{\begin{array}{cc} \sqrt{qr-r^{2}}\tan^{-1}\left(\frac{Y}{\sqrt{qr-r^{2}}}\right) & \mathrm{if}\;qr-r^{2}>0,\\ -\frac{\sqrt{r^{2}-rq}}{2}\ln\left(\frac{Y-\sqrt{r^{2}-rq}}{Y+\sqrt{r^{2}-rq}}\right) & \mathrm{if}\;qr-r^{2}<0. \end{array}\right.$$

Note that this is continuous through q=r. For q=0, we can directly compute

(17) $$L=\frac{1}{\sqrt{2}}\frac{\left|r\right|}{r}\ln\left(\frac{T}{T+r}\right).$$

For r=0 we have

(18) $$L=-\sqrt{2q}{\left[\frac{1}{\sqrt{T}}\right]}_{T_{i}}^{T_{f}}.$$

### 3.2. Wootters’ Distance

By using Equations (12) and (13) in (6), we obtain

(19) $$\begin{array}{ccc} W[P(x,0),P(x,t)] & = & {\left[\frac{\beta_{0}\beta }{\pi^{2}}\right]}^{\frac{1}{4}}\int dxe^{\frac{-\beta_{0}}{2}{\left(x-x_{0}\right)}^{2}-\frac{\beta }{2}{\left(x-\langle x\rangle \right)}^{2}}\\ & = & {\left[\frac{2\sqrt{\beta_{0}\beta }}{\beta_{0}+\beta }\right]}^{\frac{1}{2}}\exp\left(-\frac{\beta \beta_{0}{\left(x_{0}-\langle x\rangle \right)}^{2}}{2(\beta_{0}+\beta )}\right), \end{array}$$

where $\langle x\rangle =x_{0}e^{-\gamma t}$.

### 3.3. Kullback–Leibler Relative Entropy

By using Equations (12) and (13) in (7), we can show

(20) $$\begin{array}{ccc} K[P(x,0)|P(x,t)] & = & \int dxP\left(x,0\right)\left[\ln\sqrt{\frac{\beta_{0}}{\beta }}e^{-\beta_{0}{\left(x-x_{0}\right)}^{2}+\beta {\left(x-\langle x\rangle \right)}^{2}}\right]\\ & = & \ln\sqrt{\frac{\beta_{0}}{\beta }}+\int dxP\left(x,0\right)\left[-\beta_{0}{\left(x-x_{0}\right)}^{2}+\beta {\left(x-\langle x\rangle \right)}^{2}\right]\\ & = & \ln\sqrt{\frac{\beta }{\beta_{0}}}+\beta {\left(x_{0}-\langle x\rangle \right)}^{2}+\frac{\beta }{2\beta_{0}}-\frac{1}{2}, \end{array}$$

where $\langle x\rangle =x_{0}e^{-\gamma t}$.

### 3.4. Jensen Divergence

By using Equations (12) and (13) in (8), we obtain

(21) $$\begin{array}{ccc} J[P(x,0)|P(x,t)] & = & \left(\frac{\beta +\beta_{0}}{2}\right){\left(x_{0}-\langle x\rangle \right)}^{2}+\frac{\beta^{2}+\beta_{0}^{2}}{4\beta_{0}\beta }-\frac{1}{2}. \end{array}$$

### 3.5. Comparison

**Figure 1:**
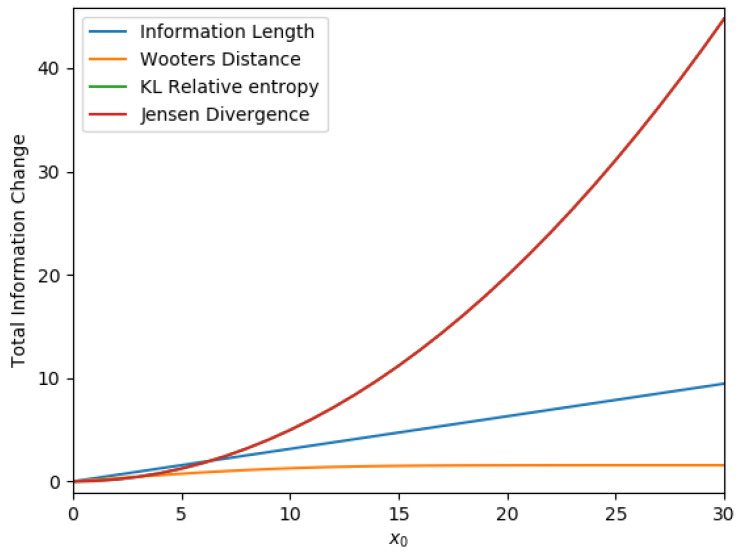
The metrics against $x_{0}$ in the long time limit for a single Ornstein–Uhlenbeck (O-U) process.

**Figure A1:**
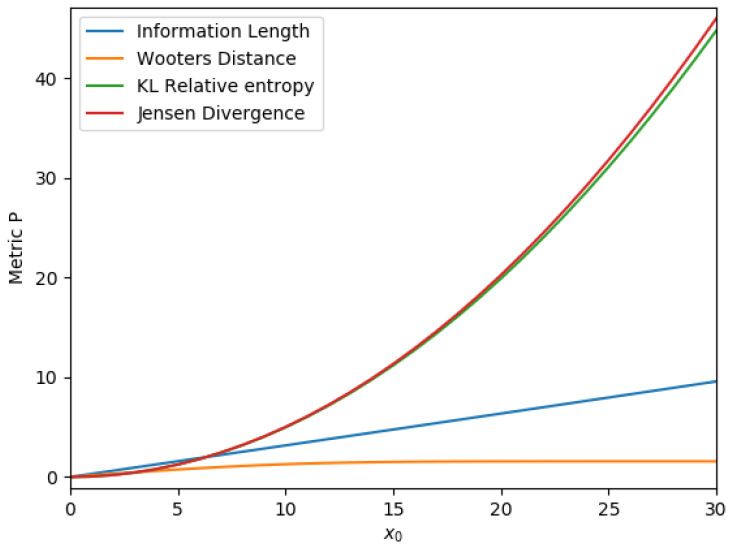
The metrics calculated by the numerical solutions to the Fokker–Planck equation by time-stepping.

Figure 1 shows the final value for each metric as we vary the initial position $x_{0}$ of the system. The total information length $L_{\infty }=L\left(t\to \infty \right)$, Wootters’ distance, K-L relative entropy and Jensen divergence against $x_{0}$ are shown in blue, orange, green and red, respectively, in the long time limit as t→∞. Note that the green and red lines are overlapped. It is notable in Figure 1 that the linear relation between the metric and the initial mean value $x_{0}$ is obtained only by the information length. That is, it is only the information length that preserves the linear geometry underlying a linear stochastic process. For all other metrics, this linear relation is lost. We show in the Appendix A that our analytical metrics in Figure 1 has a good agreement with those calculated directly from the numerical solutions to the Fokker–Planck Equation (11) by time-stepping (see Figure A1).

## 4. The Coupled O-U Process

We now consider the coupled system of equations

(22) $$\frac{\partial P_{1}}{\partial t}=\frac{\partial }{\partial x}\left[\gamma_{1}\left(x-\mu \right)P_{1}\right]+D\frac{\partial^{2}P_{1}}{\partial x^{2}}-f_{0}P_{1}+g_{0}P_{2},$$

(23) $$\frac{\partial P_{2}}{\partial t}=\frac{\partial }{\partial x}\left[\gamma_{2}\left(x-\mu \right)P_{2}\right]+D\frac{\partial^{2}P_{2}}{\partial x^{2}}+f_{0}P_{1}-g_{0}P_{2},$$

where *D* is the strength of a short-correlated Gaussian noise given by Equation (10). These equations are a pair of O-U processes, linked by coupling constants $f_{0}$ and $g_{0}$. The coupling $f_{0}$ and $g_{0}$ are due to the Dichotomous noise [25] (see Appendix B for the Langevin equation corresponding to Equations (22) and (23)).

We choose a coupled system like this one to examine the localized dynamics of these interacting sub-processes. This system could model any process for which there are two competing components relaxing to an equilibrium, like evaporation in a closed system, or a reversible chemical reaction.

Since we are mainly interested in the relaxation process from non-equilibrium initial states, we choose the different initial conditions for $P_{1}$ and $P_{2}$ while for simplicity, considering the case where $\gamma_{1}=\gamma_{2}=\gamma$ and $f_{0}=g_{0}=\epsilon$ where the Fokker–Planck Equations (22) and (23) are reduced to

(24) $$\begin{array}{ccc} \frac{\partial }{\partial t}P_{1}\left(x,t\right) & = & \frac{\partial }{\partial x}\left[\gamma \;P_{1}\left(x,t\right)\right]+D\frac{\partial^{2}}{\partial x^{2}}P_{1}\left(x,t\right)+\epsilon \left(P_{2}-P_{1}\right), \end{array}$$

(25) $$\begin{array}{ccc} \frac{\partial }{\partial t}P_{2}\left(x,t\right) & = & \frac{\partial }{\partial x}\left[\gamma \;P_{2}\left(x,t\right)\right]+D\frac{\partial^{2}}{\partial x^{2}}P_{2}\left(x,t\right)+\epsilon \left(P_{1}-P_{2}\right). \end{array}$$

Specifically, as initial conditions, we use the following different Gaussian PDFs

(26) $$\begin{array}{ccc} P_{1}\left(x,0\right) & = & \frac{1}{2}\sqrt{\frac{\beta_{10}}{\pi }}\exp\left[-\beta_{10}x^{2}\right], \end{array}$$

(27) $$\begin{array}{ccc} P_{2}\left(x,0\right) & = & \frac{1}{2}\sqrt{\frac{\beta_{20}}{\pi }}\exp\left[-\beta_{20}{\left(x-x_{0}\right)}^{2}\right]. \end{array}$$

Note that $\beta_{10}$ and $\beta_{20}$ are the initial inverse temperatures for $P_{1}$ and $P_{2}$, respectively. On the other hand, we fix the initial mean of $P_{1}$ to be zero while the initial $P_{2}$ is taken to have any arbitrary mean value $x_{0}$. We also note that as t→∞, $P_{1}$ and $P_{2}$ approach the same PDF.

To solve Equations (24) and (25), we take the Fourier transform [${\tilde{P}}_{m}\left(k,t\right)=\int dx\;e^{ikx}P_{m}\left(x,t\right)$ for m=1,2] and use the characteristic equation to recast Equations (24) and (25) as

(28) $$\begin{array}{ccc} \frac{d{\tilde{P}}_{1}}{dt} & = & -Dk^{2}{\tilde{P}}_{1}+\epsilon \left({\tilde{P}}_{2}-{\tilde{P}}_{1}\right), \end{array}$$

(29) $$\begin{array}{ccc} \frac{d{\tilde{P}}_{2}}{dt} & = & -Dk^{2}{\tilde{P}}_{2}+\epsilon \left({\tilde{P}}_{1}-{\tilde{P}}_{2}\right). \end{array}$$

Here $\frac{d}{dt}=\frac{\partial }{\partial t}+\frac{dk}{dt}\frac{\partial }{\partial x}$ is the total derivative along the characteristic defined by

(30) $$\frac{dk}{dt}=\gamma k,$$

which has the solution

(31) $$k\left(t\right)=k_{0}e^{\gamma t},$$

where $k_{0}=k\left(0\right)$ is the initial wavenumber. We solve Equations (28) and (29) in terms of new variables

(32) $$\begin{array}{ccc} {\bar{P}}_{m} & = & {\tilde{P}}_{m}\exp\left(\epsilon t+D\int_{0}^{t}dt_{1}\;k{\left(t_{1}\right)}^{2}\right), \end{array}$$

for m=1,2. The coupled Equations (28) and (29) are then simplified as

(33) $$\begin{array}{ccc} \frac{d{\bar{P}}_{1}}{dt} & = & \epsilon {\bar{P}}_{2}, \end{array}$$

(34) $$\begin{array}{ccc} \frac{d{\bar{P}}_{2}}{dt} & = & \epsilon {\bar{P}}_{1}. \end{array}$$

We write down the solutions to Equations (33) and (34) using the two constants *a* and *b*

(35) $$\begin{array}{ccc} {\bar{P}}_{1} & = & ae^{\epsilon t}+be^{-\epsilon t}, \end{array}$$

(36) $$\begin{array}{ccc} {\bar{P}}_{2} & = & ae^{\epsilon t}-be^{-\epsilon t}. \end{array}$$

To determine *a* and *b*, we take the Fourier transform of the initial conditions Equations (26) and (27) to obtain

(37) $$\begin{array}{ccc} {\tilde{P}}_{1}\left(0\right) & = & \frac{1}{2}e^{-\frac{k_{0}^{2}}{4\beta_{2}}}, \end{array}$$

(38) $$\begin{array}{ccc} {\tilde{P}}_{2}\left(0\right) & = & \frac{1}{2}e^{ik_{0}x-\frac{k_{0}^{2}}{4\beta_{1}}}. \end{array}$$

Thus, evaluating Equation (32) for m=1,2 at t=0 and equating them to Equations (37) and (38), we find

(39) $$\begin{array}{ccc} a & = & \frac{1}{2}{\tilde{P}}_{1}\left(0\right)+\frac{1}{2}e^{-ik_{0}x}{\tilde{P}}_{2}\left(0\right), \end{array}$$

(40) $$\begin{array}{ccc} b & = & \frac{1}{2}{\tilde{P}}_{1}\left(0\right)-\frac{1}{2}e^{-ik_{0}x}{\tilde{P}}_{2}\left(0\right). \end{array}$$

On the other hand, using Equation (31) in Equation (32), we write ${\tilde{P}}_{m}$ (m=1,2) in terms of ${\bar{P}}_{m}$ as

(41) $${\tilde{P}}_{m}=\exp\left[-\epsilon t-\frac{D}{2\gamma }\left(1-e^{-2\gamma t}\right)k^{2}\right]\;{\bar{P}}_{m}.$$

Finally, taking the inverse Fourier transform [$P_{m}\left(x,t\right)=\frac{1}{2\pi }\int dx\;e^{-ikx}\;{\tilde{P}}_{m}\left(x,t\right)$ for m=1,2] and performing several Gaussian integrals, we obtain

(42) $$\begin{array}{ccc} P_{1}\left(x,t\right) & = & \frac{1}{4}\left[\sqrt{\frac{\beta_{1}}{\pi }}\left(1+e^{-2\epsilon t}\right)e^{-\beta_{1}x^{2}}+\sqrt{\frac{\beta_{2}}{\pi }}\left(1-e^{-2\epsilon t}\right)e^{-\beta_{2}{\left(x-e^{-\gamma t}x_{0}\right)}^{2}}\right], \end{array}$$

(43) $$\begin{array}{ccc} P_{2}\left(x,t\right) & = & \frac{1}{4}\left[\sqrt{\frac{\beta_{1}}{\pi }}\left(1-e^{-2\epsilon t}\right)e^{-\beta_{1}x^{2}}+\sqrt{\frac{\beta_{2}}{\pi }}\left(1+e^{-2\epsilon t}\right)e^{-\beta_{2}{\left(x-e^{-\gamma t}x_{0}\right)}^{2}}\right], \end{array}$$

where

(44) $$\frac{1}{2\beta_{m}}=\frac{e^{-2\gamma t}}{2\beta_{m0}}+\frac{D}{\gamma }\left(1-e^{-2\gamma t}\right),$$

for m=1,2. We can check that at t=0, Equations (42) and (43) recover Equations (26) and (27). On the other hand, in the limit of t→∞, Equations (42) and (43) give us

(45) $$\begin{array}{c} P_{m}\left(x,t\right)=\frac{1}{2}\sqrt{\frac{\beta_{m}\left(t\to \infty \right)}{\pi }}e^{-\beta_{m}\left(t\to \infty \right)x^{2}}, \end{array}$$

which is the stationary solution to a single O-U process where $\beta_{m}\left(t\to \infty \right)=\frac{\gamma }{2D}$. We note that the total PDF $P=P_{1}+P_{2}$ is the solution of this single O-U process with the initial condition given by the sum of Equations (26) and (27).

Using these analytical solutions in Equations (42)–(44), we present the different metrics in Equations (1) and (5)–(8) in Section 5.

## 5. Results for the Coupled O-U Process

For the illustration in this section, we show results for the fixed parameter values γ=0.1, D=1 and ϵ=0.5. We recall from Section 4 that for these parameter values, in equilibrium (see Equation (44)), $\beta_{1}=\beta_{2}=\beta \left(t\to \infty \right)=\frac{\gamma }{2D}=0.05$ while the mean values in Equation (45) are zero for both $P_{1}$ and $P_{2}$. For comparing metrics, we consider the case where $P_{1}$ is initially in the final equilibrium with the zero mean value and inverse temperature $\beta_{10}=0.05$. On the other hand, $P_{2}$ at t=0 is taken to have either different mean values $x_{0}$ or different inverse temperatures $\beta_{20}$. Here, we present results obtained by using analytical solutions in Section 4 only. (See Appendix A for the numerical solutions and comparison with the analytical solutions.)

### 5.1. Varying $\beta_{20}$

We first examine how the system changes when varying the initial inverse temperature $\beta_{20}$ of $P_{2}$ for the fixed zero mean position ($x_{0}=0$) (the equilibrium value). We investigate what changes are detected by each metric.

**Figure 2:**
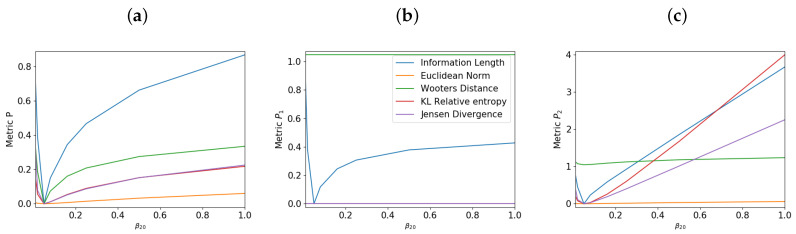
Behavior of the metrics for varying $\beta_{20}$ for the overall system in panel (**a**) and components $P_{1}$ in panel (**b**) and $P_{2}$ in panel (**c**).

Figure 2 shows the total information length (in blue), Euclidean norm (in orange), Wootters’ distance (in green), K-L relative entropy (in red), and Jensen divergence (in purple) against $\beta_{20}$ in the long time limit as t→∞. Panels (a), (b) and (c) are for the overall system *P*, and the components $P_{1}$ and $P_{2}$, respectively. Since $P_{1}$ is initially chosen to be in its equilibrium state, the behavior for the overall system *P* in panel (a) is more similar to $P_{2}$ in panel (c) than $P_{1}$ in panel (b). What is prominent in both panels (a) and (c) is the presence of the distinct minimum in the information length around $\beta_{20}=0.05$. This is because the final equilibrium has the inverse temperature 0.05, demonstrating that the total information also maps out the underlying attractor structure when varying $\beta_{20}$, taking its minimum value at the equilibrium state (reminiscent of the results for the single O-U process above and previous works [13,16,18]). The minimum value around $\beta_{20}=0.05$ is also observed for other metrics (apart from the Euclidean norm) although less pronounced than the total information length. In fact, for $\beta_{20}=\beta_{10}=\beta \left(t\to \infty \right)$, there is no temporal change in both $P_{1}$ and $P_{2}$, so all the metrics apart from the Wootters’ one take the value of zero. Also, of interest is an almost linear increase in the total information length for $P_{2}$ in panel (c) as $|\beta_{20}-0.05|$ increases.

Now, what is happening to $P_{1}$ which starts with the equilibrium state ($\beta_{10}=0.05$ and zero mean value)? While Equation (44) shows that $\beta_{1}=\beta_{10}=\gamma /2D=0.05$ for all time, the actual PDF $P_{1}$ in Equation (42) changes with time due to its coupling to $P_{2}$ via ϵ. That is, $P_{1}$ changes over time, initially deviating from the equilibrium state due to the interaction with $P_{2}$ and then finally relaxing back to the final equilibrium state. Associated with this time evolution of $P_{1}$ is the information change which can be measured by different metrics. However, Figure 2b shows that for $P_{1}$, it is only information length that detects a noticeable difference in the information change as $\beta_{20}$ changes. Furthermore, the information length for $P_{1}$ takes the minimum value for the equilibrium value of $\beta_{20}=0.05$, as was the case for $P_{2}$. This result demonstrates that the information length is sensitive to the evolution of of the component $P_{1}$ (a subsystem).

**Figure 3:**
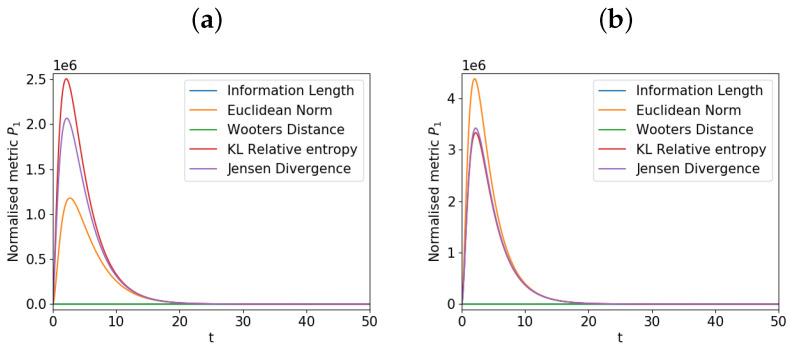
The metrics against time for $P_{1}$ around $\beta_{20}=0.05$. $\beta_{20}=0.02$ in panel (**a**) and $\beta_{20}=0.08$ in panel (**b**). The Y-axis scaling on the panels is $10^{6}$.

To investigate the evolution of the metrics for the $P_{1}$ around $\beta_{20}$, we show in Figure 3 how the metrics evolve over time for the component $P_{1}$ for the two different values of $\beta_{20}=0.02$ and 0.08 near the equilibrium value $\beta_{20}=0.05$. Panel a) is for $\beta_{20}=0.02<0.05$ and panel b) is for $\beta_{20}=0.08>0.05$. The different metrics are denoted by using the same color as used in Figure 2. This small deviation of $\beta_{20}$ from the equilibrium value induces a small change in $P_{1}$ over time due to the coupling to $P_{2}$, as noted above. However, in panels a) and b), we see a significant change in the Euclidean norm, K-L relative entropy, and Jensen divergence in time, with a large increase before settling down to a lower value. In comparison, the information length shows no such spike. These spikes are caused by the deviation of $P_{1}$ from its initial equilibrium state due to the coupling to $P_{2}$ before settling into the equilibrium state as $P_{2}$ approaches the equilibrium. What is interesting is that the information length does not show such a spike since it measures the local information change; this would thus be the more sensible view in this instance since the change in the component $P_{1}$ is small compared to its width (uncertainly). Furthermore, such spikes do not appear for $P_{2}$ nor *P* (results not shown) since $P_{2}$ starting from a non-equilibrium state monotonically approaches its equilibrium.

### 5.2. Varying $x_{0}$

**Figure 4:**
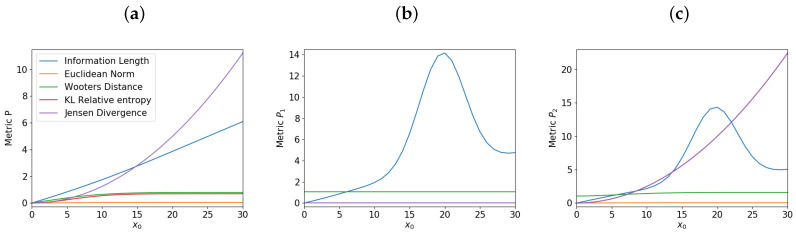
Behavior of the metrics for varying $x_{0}$ for the overall system *P* in panel (**a**), $P_{1}$ in (**b**), and $P_{2}$ in (**c**).

We now fix $\beta_{20}=0.05$ and vary the initial mean position $x_{0}$ of $P_{2}$ to examine how metrics depend on $x_{0}$, as we have done for the single O-U process in Section 3. Figure 4 shows the total information change for each metric for different values of $x_{0}$, for *P*, $P_{1}$ and $P_{2}$ in panels (a), (b), and (c) respectively. Specifically, Figure 4a shows that for *P*, the total information length against $x_{0}$ is linear, capturing the linearity of the system as expected from the single O-U process. None of the other metrics are capable of showing the linear relationship in the same way. On the other hand, Figure 4b,c shows an interesting non-monotonic dependence of the information length on $x_{0}$.

**Figure 5:**
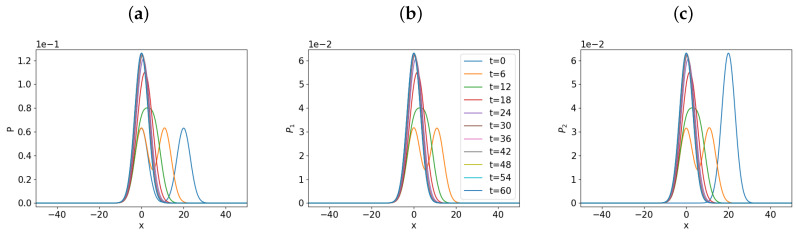
Time-dependent partial differential equations (PDFs) for *P* in panel (**a**), for $P_{1}$ in panel (**b**) and for $P_{2}$ in panel (**c**); $x_{0}=20$.

To understand this, we show in Figure 5 the time evolution of *P*, $P_{1}$, and $P_{2}$ in panels (a), (b), and (c), respectively, by using $x_{0}=20$. Of interest is that the evolution of $P_{1}$ and $P_{2}$ in Figure 5b,c involves the formation of the two peaks from the initial one peak, followed by merging of these two peaks into one peak as a system settles into the equilibrium. This formation of the two peaks is due to the interaction between $P_{1}$ and $P_{2}$ when they are initially widely separated for a sufficiently large $x_{0}⪆20$. The formation of two PDFs peaks for $x_{0}⪆20$ leads to the maximum in the total information length around $x_{0}=20$ in Figure 4b,c. Specifically, the formation of the two peaks in $P_{1}$ and $P_{2}$ shown in Figure 5b,c takes place when the two peak are a full PDF width apart, and facilitates broadening of both PDFs in the relaxation process. As $x_{0}$ is further increased from 20, $P_{1}$ and $P_{2}$ form two peaks which are more widely separated, leading to $P_{1}$ and $P_{2}$ becoming effectively broader. This in turn reduces the information length (as $x_{0}$ increases further from $x_{0}=20$) since large fluctuations (uncertainty) associated with a broad PDF reduces the information length. Again, the information length is the only measure which detects the difference in the overall information change for $P_{1}$ due to its sensitive dependence on the local evolution of a system.

## 6. Conclusions

When searching for a way to quantify the information change in a given dynamical system, our choices are many and varied. Our aim here was to show the power of the information length L when compared with some of the more popular methods of measuring information change. Utilizing the O-U, we compared several relative-entropy formulations with our information length to investigate what each could reveal about the dynamics of system.

Specifically, we investigated the relaxation problem given by a single and coupled O-U process and compared the information length L with K-L relative entropy, Jensen divergence, Wootters’ distance, and Euclidean norm. By measuring the total information length in the long time limit, we showed that L was unique in detecting the linear spatial relationship between the total information change and the initial position of a PDF. In the coupled O-U process, the information length was shown to be the most effective in detecting changes in the components of the system even when the others would detect almost nothing in one of the components. In particular, when $P_{1}$ started with an equilibrium state with the zero mean value, the formation of the two peaks of $P_{1}$ (or $P_{2}$) from an initial one peak $P_{1}$ (or $P_{2}$) and the merging of the two peaks into one peak as a system settled into equilibrium was detected only by the information length with its intriguing non-monotonic dependence on $x_{0}$ (the mean value of $P_{2}$). This underscores the sensitivity of the information length on the evolution of subsystems.

Future work will include the study of a system with multiple attractor positions for the system or how the system behaves when changing the position of the attractor. It would also be interesting to examine the case where the coupling parameters $f_{0}$ and $g_{0}$ are not constant, but are functions of time. This could result in a periodic equilibrium where the PDF varies between 2 or more unstable states. This could represent physical systems like reversible chemical reactions, or even fluctuating financial markets. It would also be of interest to investigate implication of the information length for the deep neural network [26], in particular, to elucidate the role of geodesic along which the information length is minimized [27].

## Figures and Tables

**Figure A2:**
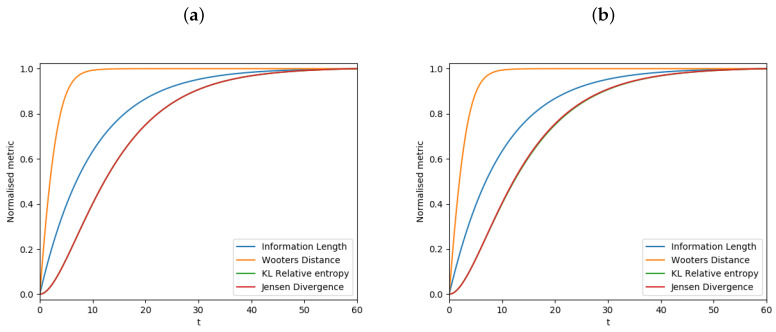
Comparing the evolution of the metrics with $x_{0}=30$. Panels (**a**) and (**b**), respectively, are obtained by using the analytical and numerical solutions to the Fokker–Planck equation.

## Appendix A

In this appendix, we show the numerical solutions to the Fokker-Planck equation by time-stepping and metrics using these numerical solutions for the O-U process and the coupled O-U processes.

### Appendix A.1. The O-U Process

We use the finite difference method to approximate the partial derivatives from Equation (11), where *h* is our time-step, *s* is our spatial step, *i* is the current time step, and *j* is the current spatial step.

(A1) $$\frac{P_{i+1}^{j}-P_{i}^{j}}{h}=\gamma \frac{\left(x_{j}-x_{0}\right)\left[P_{i}^{j+1}-P_{i}^{j}\right]}{s}+D\frac{P_{i}^{j+1}-2P_{i}^{j}+P_{i}^{j-1}}{s^{2}}.$$

Then using a second-order Runga-Kutta Method, we create a time-stepped solution. Specifically, we first create an intermediate value $\tilde{P}$ by

(A2) $${\left(\tilde{P}\right)}_{i+1}^{j}=P_{i}^{j}+hf\left(t_{i},P_{i}^{j}\right),$$

where

(A3) $$f\left(t_{i},P_{i}^{j}\right)=\gamma \frac{\left(x_{j}-x_{0}\right)\left[P_{i}^{j+1}-P_{i}^{j}\right]}{s}+D\frac{P_{i}^{j+1}-2P_{i}^{j}+P_{i}^{j-1}}{s^{2}},$$

which is the time-gradient for the system. Here, $t_{i}$ is the current time value. Then we use the intermediate value to calculate the next time step value, $P_{i+1}^{j}$, using the formula

(A4) $$P_{i+1}^{j}=P_{i}^{j}+\frac{h}{2}\left[f\left(t_{i},P_{i}^{j}\right)+f{\left(t_{i+1},\tilde{P}\right)}_{i+1}^{j}\right)].$$

For simplicity, we set D=1. This is to consider the case of the one parameter system, as the O-U process can always be arranged to combine γ and *D* into a single parameter. For our stepping, we use h=0.04 and s=0.1. We check on the convergence of our solutions by reducing *h* and *s*. We use the initial condition given by Equation (13). All solutions are coded in pythonuni We present the numerically computed metrics from the time-stepping in Figure A1, which shows the final value for each metric as we vary the initial position of the system, $x_{0}$. Figure A1 is quite similar to the analytically calculated metrics shown in Figure 1, demonstrating a good agreement between the analytical and numerical solutions.

To test the accuracy of our time-stepped model further, Figure A2 compares the time evolution of different metrics for the largest $x_{0}$ value, i.e., the largest perturbation from equilibrium in our testing, with analytic and time-stepped solutions on the left and right respectively. The agreement between the analytical and numerical solutions is observed to be quite good.

### Appendix A.2. Coupled O-U Process

We solve (22) and (23) numerically by time-stepping, using the same method as for the single equation case. Our discrete versions of (22) and (23) become

(A5) $$\frac{{\left(P_{1}\right)}_{i+1}^{j}-{\left(P_{1}\right)}_{i}^{j}}{h}=\gamma_{1}\frac{\left(x_{j}-\mu_{1}\right)\left[{\left(P_{1}\right)}_{i}^{j+1}-{\left(P_{1}\right)}_{i}^{j}\right]}{s}+D_{1}\frac{{\left(P_{1}\right)}_{i}^{j+1}-2{\left(P_{1}\right)}_{i}^{j}+{\left(P_{1}\right)}_{i}^{j-1}}{s^{2}}-\epsilon {\left(P_{1}\right)}_{i}^{j}+\epsilon {\left(P_{2}\right)}_{i}^{j}$$

(A6) $$\frac{{\left(P_{2}\right)}_{i+1}^{j}-{\left(P_{2}\right)}_{i}^{j}}{h}=\gamma_{2}\frac{\left(x_{j}-\mu_{2}\right)\left[{\left(P_{2}\right)}_{i}^{j+1}-{\left(P_{2}\right)}_{i}^{j}\right]}{s}+D_{2}\frac{{\left(P_{2}\right)}_{i}^{j+1}-2{\left(P_{2}\right)}_{i}^{j}+{\left(P_{2}\right)}_{i}^{j-1}}{s^{2}}+\epsilon {\left(P_{1}\right)}_{i}^{j}-\epsilon {\left(P_{2}\right)}_{i}^{j},$$

where *h* is our time-step, and *s* is our spatial-step. We then use Huen’s method to approximate the time-evolution for the system. So our final numerical model becomes

(A7) $${\left(\tilde{P_{1}}\right)}_{i+1}^{j}={\left(P_{1}\right)}_{i}^{j}+hf_{1}\left(t_{i},{\left(P_{1}\right)}_{i}^{j},{\left(P_{2}\right)}_{i}^{j}\right),$$

(A8) $${\left(\tilde{P_{2}}\right)}_{i+1}^{j}={\left(P_{2}\right)}_{i}^{j}+hf_{2}\left(t_{i},{\left(P_{1}\right)}_{i}^{j},{\left(P_{2}\right)}_{i}^{j}\right),$$

(A9) $${\left(P_{1}\right)}_{i+1}^{j}={\left(P_{1}\right)}_{i}^{j}+\frac{h}{2}\left[f_{1}\left(t_{i},{\left(P_{1}\right)}_{i}^{j},{\left(P_{2}\right)}_{i}^{j}\right)+f_{1}\left(t_{i+1},{\left(\tilde{P_{1}}\right)}_{i+1}^{j},{\left(\tilde{P_{2}}\right)}_{i+1}^{j}\right)\right],$$

(A10) $${\left(P_{2}\right)}_{i+1}^{j}={\left(P_{2}\right)}_{i}^{j}+\frac{h}{2}\left[f_{2}\left(t_{i},{\left(P_{1}\right)}_{i}^{j},{\left(P_{2}\right)}_{i}^{j}\right)+f_{2}\left(t_{i+1},{\left(\tilde{P_{1}}\right)}_{i+1}^{j},{\left(\tilde{P_{2}}\right)}_{i+1}^{j}\right)\right],$$

where

(A11) $$f_{1}\left(t_{i},{\left(P_{1}\right)}_{i}^{j},{\left(P_{2}\right)}_{i}^{j}\right)=\gamma_{1}\frac{\left(x_{j}-\mu_{1}\right)\left[{\left(P_{1}\right)}_{i}^{j+1}-{\left(P_{1}\right)}_{i}^{j}\right]}{s}+D_{1}\frac{{\left(P_{1}\right)}_{i}^{j+1}-2{\left(P_{1}\right)}_{i}^{j}+{\left(P_{1}\right)}_{i}^{j-1}}{s^{2}}-\epsilon {\left(P_{1}\right)}_{i}^{j}+\epsilon {\left(P_{2}\right)}_{i}^{j}$$

(A12) $$f_{2}\left(t_{i},{\left(P_{1}\right)}_{i}^{j},{\left(P_{2}\right)}_{i}^{j}\right)=\gamma_{2}\frac{\left(x_{j}-\mu_{2}\right)\left[{\left(P_{2}\right)}_{i}^{j+1}-{\left(P_{2}\right)}_{i}^{j}\right]}{s}+D_{2}\frac{{\left(P_{2}\right)}_{i}^{j+1}-2{\left(P_{2}\right)}_{i}^{j}+{\left(P_{2}\right)}_{i}^{j-1}}{s^{2}}-\epsilon {\left(P_{2}\right)}_{i}^{j}+\epsilon {\left(P_{1}\right)}_{i}^{j},$$

and ${\left(\tilde{P_{k}}\right)}_{i+1}^{j}$ are the intermediate values. Here we set $D_{1}=D_{2}=1$ and use h=0.04 and s=0.1.

We give our system components a Gaussian initial condition, given by

(A13) $${\left(P_{1}\right)}_{0}^{j}=\frac{1}{2}\sqrt{\frac{\beta_{1}}{\pi }}e^{-\beta_{1}{\left(x_{j}-\nu_{1}\right)}^{2}},$$

(A14) $${\left(P_{2}\right)}_{0}^{j}=\frac{1}{2}\sqrt{\frac{\beta_{2}}{\pi }}e^{-\beta_{2}{\left(x_{j}-\nu_{2}\right)}^{2}},$$

where $\beta_{1}$ and $\beta_{2}$ are the inverse temperature for $P_{1}$ and $P_{2}$ respectively, and $\nu_{1}$ and $\nu_{2}$ are their initial mean positions.

When varying the system parameters, we fix the parameters for $P_{1}$ at equilibrium ($\nu_{1}=0$, $\beta_{1}=0.05$, $\gamma_{1}=0.1$) and vary the parameters for $P_{2}$ ($\nu_{2}$, $\beta_{2}$). The choice between fixing $P_{1}$ or $P_{2}$ is arbitrary. We solve the Fokker–Planck equations for the coupled O-U process (Equations (22) and (23)) by time-stepping and then numerically calculated the metrics. These are shown to have a good agreement with the results using the analytic formulation of the PDF (Equations (42) and (43)) in Section 5.

## Appendix B

The Langevin equation corresponding to Equations (22) and (23) is given by

(A15) $$\begin{array}{ccc} \partial_{t}x & = & -(x-\mu )\eta (t)+\xi . \end{array}$$

Here, ξ is the Gaussian noise given by Equation (10), and η(t) is the dichotomous Markov noise (DMN) [25,28] which takes the two value $\left\{\gamma_{1},\gamma_{2}\right\}$, with constant transition rates $k_{\pm }=f_{0},g_{0}$ between the two states. A pure DMN case is governed by

(A16) $$\begin{array}{ccc} \partial_{t}x & = & \eta (t), \end{array}$$

and the waiting time $\tau_{\pm }$ in the two states are exponentially-distributed stochastic variable, with the probability

(A17) $$P\left(\tau_{\pm }\right)=k_{\pm }e^{-k_{\pm }\tau_{\pm }}.$$

Note that η(t) is not a white noise, but has the exponentially decaying correlation function as

(A18) $$\langle \eta \left(t\right)\eta \left(t^{\prime }\right)\rangle =\frac{Q}{\tau_{c}}\exp\left(-\frac{|t-t^{\prime }|}{\tau_{c}}\right).$$

Here, $Q=k_{+}k_{-}\tau_{c}^{3}{\left(\gamma_{1}+\gamma_{2}\right)}^{2}$ and $\tau_{c}=\frac{1}{k_{+}+k_{-}}$ is the characteristic relaxation time to the stationary state of the DMN. For the DMN, due to a finite correlation time, an exact, analytical time-dependent PDF, in general, is unavailable. For instance, the pure DMN case given by Equation (A17) leads to a coupled linear Fokker–Planck equation (e.g., see Equations (9) and (10) in [28]), but a resulting equation for the total PDF is a differential and integral equation in time (see, e.g., Equation (11) in [28]).

## References

[1] Zamir R. (1998). A proof of the Fisher information inequality via a data processing argument. IEEE Trans. Inf. Theory.

[2] Gibbs A.L., Su F.E. (2002). On choosing and bounding probability metrics. Int. Stat. Rev..

[3] Jordan R., Kinderlehrer D., Otto F. (1998). The variational formulation of the Fokker–Planck equation. SIAM J. Math. Anal..

[4] Lott J. (2008). Some geometric calculations on Wasserstein space. Commun. Math. Phys..

[5] Takatsu A. (2011). Wasserstein geometry of Gaussian measures. Osaka J. Math..

[6] Otto F., Villani C. (2000). Generalization of an Inequality by Talagrand and Links with the Logarithmic Sobolev Inequality. J. Funct. Anal..

[7] Costa S., Santos S., Strapasson J. (2015). Fisher information distance. Discret. Appl. Math..

[8] Frieden B.R. (2004). Science from Fisher Information.

[9] Wootters W.K. (1981). Statistical distance and Hilbert space. Phys. Rev. D.

[10] Kullback S. (1951). Letter to the Editor: The Kullback-Leibler distance. Am. Stat..

[11] Kowalski A.M., Martin M.T., Plastino A., Rosso O.A., Casas M. (2011). Distances in Probability Space and the Statistical Complexity Setup. Entropy.

[12] Information Length. https://encyclopedia.pub/238.

[13] Heseltine J., Kim E. (2016). Novel mapping in non-equilibrium stochastic processes. J. Phys. A.

[14] Kim E. (2018). Investigating Information Geometry in Classical and Quantum Systems through Information Length. Entropy.

[15] Kim E., Lewis P. (2018). Information length in quantum systems. J. Stat. Mech..

[16] Kim E., Hollerbach R. (2017). Signature of nonlinear damping in geometric structure of a nonequilibrium process. Phys. Rev. E.

[17] Kim E., Hollerbach R. (2017). Geometric structure and information change in phase transitions. Phys. Rev. E.

[18] Hollerbach R., Dimanche D., Kim E. (2018). Information geometry of nonlinear stochastic systems. Entropy.

[19] Hollerbach R., Kim E., Mahi Y. (2019). Information length as a new diagnostic in the periodically modulated double-well model of stochastic resonance. Physica A.

[20] Kim E., Hollerbach R. (2016). Time-dependent probability density function in cubic stochastic processes. Phys. Rev. E.

[21] Nicholson S.B., Kim E. (2015). Investigation of the statistical distance to reach stationary distributions. Phys. Lett. A.

[22] Matey A., Lamberti P.W., Martin M.T., Plastron A. (2005). Wotters’ distance resisted: A new distinguishability criterium. Eur. Rhys. J. D.

[23] Risken H. (1996). The Fokker-Planck Equation: Methods of Solution and Applications.

[24] Klebaner F. (2012). Introduction to Stochastic Calculus with Applications.

[25] Bena I. (2006). Dichotomous Markov Noise: Exact results for out-of-equilibrium systems (a brief overview). Int. J. Mod. Phys. B.

[26] Shwartz-Ziv R., Tishby N. (2017). Opening the Black Box of Deep Neural Networks via Information. arXiv.

[27] Kim E., Lee U., Heseltine J., Hollerbach R. (2016). Geometric structure and geodesic in a solvable model of nonequilibrium process. Phys. Rev. E.

[28] Van Den Brock C. (1983). On the relation between white shot noise, Gaussian white noise, and the dichotomic Markov process. J. Stat. Phys..

